# Advances in Magnetic Nanocomposites: A New Open Special Issue in *Materials*

**DOI:** 10.3390/ma15196905

**Published:** 2022-10-05

**Authors:** Anatoly B. Rinkevich, Dmitry V. Perov

**Affiliations:** M.N. Miheev Institute of Metal Physics UB RAS, Sofia Kovalevskaya St., 18, 620108 Ekaterinburg, Russia

**Keywords:** physical and chemical properties of nanostructures, technological applications of nanocomposites, carbon-containing materials and structures, ferromagnetic nanocomposites, microwave properties, spintronics

Carbon-based nanomaterials are crucial for most branches of modern technology. Several studies have been devoted to composites containing graphene and nanotubes, as well as ferromagnetic particles. However, magnetic interactions and their effect on microwave phenomena have not been studied in detail, and the main focus is on the optimization of radio-absorbing properties. A review of microwave absorption in composites filled with carbonaceous particles was presented in [1].

Recently, magnetic nanoparticles, nanocomposites, and nanostructures have attracted extensive attention due to their technological applications such as microwave and spintronic devices, magnetic recording media, microwave absorbers and electromagnetic interference shielding, drug-delivery, magnetic resonance imaging, magnetic refrigeration, and super-capacitors [2,3]. Magnetic nanocomposites are among these heterogeneous nanosized systems. They demonstrate specific properties conditioned by the influence of interfaces which secure their multifunctional applications. Describing physical and chemical properties of advanced nanocomposites constitutes an essential and difficult scientific task.

The fundamentals of the magnetism of microsized particles are laid down in the monograph [1]. The current state of the problems of magnetic nanocomposites is expounded in the monograph [4]. A theoretical and experimental study that clarified the features of ferromagnetic resonance in magnetic nanocomposites was carried out in [3,5]. Among the numerous practical uses of magnetic nanocomposites and nanoparticles, we note the development of sensitive sensor elements [6] and promising applications in medicine [7].

This Special Issue of *Materials*, entitled “Advances in Magnetic Nanocomposites”, aims to describe recent developments in advanced magnetic nanosized magnetic materials with detailed explanations of the structural, physical, and chemical characteristics as well as their synthesis and characterization. The papers presented here report on the properties and applications of multifunctional magnetic nanocomposites consisting of different matrices embedded or decorated by different magnetic nanoparticles of various structures, from monophase to core–shell, and nanowires, cluster-like nanostructures, carbon-containing materials and structures, as well as multilayer systems such as superlattices.
